# Glabridin exhibits potent inhibitory effects against *Toxoplasma gondii *in vitro and in vivo

**DOI:** 10.1186/s13071-024-06610-0

**Published:** 2024-12-18

**Authors:** Lu Wang, Bintao Zhai, Chen Wang, Hany M. Elsheikha, Haiting Guo, Xiao-Nan Zheng, Chun‑Xue Zhou, Xing-Quan Zhu

**Affiliations:** 1 Laboratory of Parasitic Diseases, College of Veterinary Medicine, Shanxi Agricultural University, Taigu, Shanxi Province 030801 People’s Republic of China; 2 Key Laboratory of Veterinary Pharmaceutical Development, Lanzhou Institute of Husbandry and Pharmaceutical Sciences, Chinese Academy of Agricultural Sciences, Ministry of Agriculture and Rural Affairs, Lanzhou, Gansu Province 730050 People’s Republic of China; 3 Faculty of Medicine and Health Sciences, School of Veterinary Medicine and Science, University of Nottingham, Sutton Bonington Campus, Loughborough, LE12 5RD UK; 4 Guangxi Key Laboratory of Brain and Cognitive Neuroscience, College of Basic Medicine, Guilin Medical University, Guilin, Guangxi Zhuang Autonomous Region 541199 People’s Republic of China; 5 Department of Pathogen Biology, School of Basic Medical Sciences, Cheeloo College of Medicine, Shandong University, Jinan, Shandong Province 250012 People’s Republic of China

**Keywords:** *Toxoplasma gondii*, Glabridin, Metabolomics, Transcriptomics, Survival rate

## Abstract

**Background:**

*Toxoplasma gondii* is an obligate protozoan parasite capable of infecting a wide range of warm-blooded animals and humans. Current treatment options, primarily pyrimethamine and sulfadiazine, have limitations, such as high recurrence rates, long treatment durations, and limited effectiveness against *T. gondii*. There is an unmet need for novel, safe, low-toxicity, and highly effective treatments. This study aimed to evaluate the anti-*T. gondii* effects of glabridin, a natural compound derived from the roots of a widely used medicinal plant*.*

**Methods:**

The cytotoxicity of glabridin in Vero cells was assessed using a CCK-8 cell viability assay. Quantitative polymerase chain reaction (qPCR) targeting the Tg-529 gene was developed to quantify *T. gondii* and assess the inhibitory effects of glabridin on parasite proliferation. Ultrastructural changes in *T. gondii* after treatment were examined using electron microscopy. The levels of reactive oxygen species (ROS) and mitochondrial membrane potential (ΔΨm) were examined to assess the effects of glabridin on ROS levels and ΔΨm in *T. gondii* tachyzoites. Additionally, metabolomics and transcriptomics analyses were conducted to investigate the mechanisms underlying glabridin’s anti-*T. gondii* effects.

**Results:**

Glabridin exhibited low toxicity to host cells and effectively inhibited *T. gondii* invasion and proliferation in vitro in a time-dependent manner. Glabridin-treated tachyzoites exhibited significant structural alterations, along with increased ROS production and a reduction in ΔΨm. Metabolomic analysis indicated that glabridin significantly affected amino acid metabolism pathways in *T. gondii*. In vivo, glabridin treatment significantly improved survival rates in *T. gondii*-infected BALB/c mice at a dosage of 100 mg/kg.

**Conclusions:**

This study demonstrates that glabridin has potent anti-*T. gondii* effects in vitro and in vivo, likely through disruption of amino acid metabolism in the parasite. These findings highlight glabridin’s potential as a promising therapeutic agent for toxoplasmosis.

**Graphical abstract:**

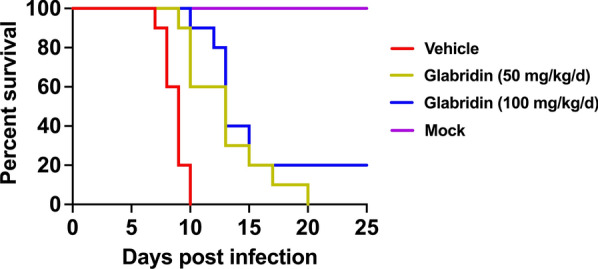

**Supplementary Information:**

The online version contains supplementary material available at 10.1186/s13071-024-06610-0.

## Background

*Toxoplasma gondii* is a zoonotic protozoan capable of infecting nearly all warm-blooded animals, affecting approximately one-third of the global human population [1–5]. The parasite is transmitted through various routes, including contaminated food, vertical transmission, organ transplantation, and blood transfusion [6]. Although often asymptomatic in immunocompetent individuals, *T. gondii* infection during pregnancy can result in severe fetal complications, such as vision loss, mental retardation, intracranial calcification, hydrocephalus, and congenital malformations [7, 8]. Additionally, *T. gondii* infections pose a serious risk to immunocompromised patients, potentially leading to life-threatening conditions [4, 9].

The complexity of *T. gondii*’s life cycle, involving both sexual and asexual reproduction, presents challenges for developing effective treatments. Sexual reproduction occurs exclusively in the intestinal epithelium of cats, the definitive hosts, while asexual reproduction can occur in a range of nucleated cells across various intermediate hosts, including humans [5]. Although some medications are available, they are often associated with limitations, such as prolonged treatment duration, toxicity, and incomplete parasite eradication, especially in immunocompromised individuals [3, 10, 11]. This underscores the urgent need for safe, effective, and low-toxicity treatments for toxoplasmosis.

Natural products have emerged as a promising avenue in the search for novel anti-*T. gondii* therapies. Interestingly, 70% of the 1562 new drugs approved between 1981 and 2014 were derived from natural sources [12]. Traditional Chinese medicine, which is rich in biologically active compounds with low toxicity, offers a valuable resource for discovering new anti-*T. gondii* agents [13]. Glabridin, an isoflavonoid extracted from the roots of *Glycyrrhiza glabra*, a commonly used medicinal plant, has shown broad pharmacological activities, including antioxidant [14, 15], anti-inflammatory [16, 17], immunomodulatory [18], and anti-tumor effects [19]. Classified as Generally Recognized As Safe (GRAS) by the USFDA, glabridin demonstrated potential anti-*T. gondii* activity in our preliminary screenings, though its precise efficacy and mechanism of action remain unclear.

Recent advancements in transcriptomics and metabolomics provide powerful tools for elucidating the mechanisms of action of bioactive compounds. Transcriptomics reveals gene expression changes under specific conditions, while metabolomics profiles small molecules within a biological system, allowing identification of potential drug targets and metabolic pathways [20–22]. Integrated transcriptomic and metabolomic analyses have previously uncovered metabolic disruptions caused by *T. gondii* infection and the effects of treatments, such as sodium sulfadiazine, in restoring metabolic balance [23, 24].

In this study, we assessed the anti-*T. gondii* activity of glabridin in vivo using a BALB/c mouse model and explored its mechanism of action through transcriptomic and metabolomic profiling of glabridin-treated *T. gondii* tachyzoites in vitro. Our findings highlight glabridin’s potential as a lead compound for toxoplasmosis treatment and contribute valuable insights to the field of protozoan drug development.

## Methods

### Mice

Eight-week-old female BALB/c mice (18–20 g) were housed under standard conditions at a temperature of 23–25 °C, relative humidity of 40–70%, and 12-h light/dark cycle. The mice were kept in well-ventilated cages with access to sterilized water and food ad libitum.

### Cells and parasites

Vero cells were cultured in DMEM supplemented with 10% FBS, 10,000 U/ml penicillin, and 10,000 µg/ml streptomycin and incubated at 37 °C with 5% CO_2_. Tachyzoites of the *T. gondii* RH strain were maintained in Vero cell monolayers and grown in DMEM containing penicillin, streptomycin, and 2% FBS. When ~80% of the tachyzoites had egressed from the cells, they were purified by aspirating the cell suspension twice through a 27G needle to disrupt the host cells. The suspension was then centrifuged at 200×*g* for 5 min, filtered through a 5-μm membrane, and centrifuged again at 1500×*g* for 10 min.

### Cytotoxicity of glabridin on Vero cells

The cytotoxicity of glabridin on Vero cells was evaluated using the Cell Counting Kit-8 (CCK-8) assay to determine the appropriate dosing concentrations. Vero cells were seeded in 96-well plates at 5 × 10^4^ cells per well and incubated for 8 h to form monolayers. Glabridin (MedChemExpress, Shanghai, China) was diluted to final concentrations of 2.5, 5, 7.5, 10, and 20 µg/ml in DMEM with 1% FBS. Control wells included 100 µl of DMEM as the blank control and 100 µl of DMEM with Vero cells as the negative control. After 24 h of treatment, 100 µl of 10% CCK-8 solution was added to each well, and the plates were incubated for an additional hour. Absorbance was measured at 450 nm using a microplate reader.

### Parasite burden assessment by absolute quantitative polymerase chain reaction (qPCR)

An absolute qPCR approach was used to assess the parasite burden. Genomic DNA (gDNA) was extracted from samples to evaluate parasite replication. The Tg-529 gene was targeted using specific primers: forward 5′-CGCTGCAGGGAGGAAGACGAAAGTTG-3′ and reverse 5′-CGCTGCAGACAGAGTGCATCTGGATT-3′ [25]. Standard curves were generated using plasmids containing the Tg-529 gene. qPCR was performed with ChamQ Universal SYBR qPCR Master Mix (Vazyme, Nanjing, China) according to the manufacturer’s instructions, with reactions conducted on the QuantStudio™ 5 system.

### Impact of glabridin pre-treatment on *T. gondii* infection in vitro

To determine whether pre-treatment of *T. gondii* with glabridin affects its ability to infect host cells, RH tachyzoites were incubated at 4 °C with either 5 µg/ml glabridin or 0.1% DMSO (vehicle) for 6, 12, or 24 h. After pre-treatment, the parasites were used to infect Vero cells at a multiplicity of infection (MOI) of 1 for 24 h. The effect of glabridin on parasite growth was assessed by measuring parasite replication using an absolute qPCR assay, as described above. This experiment seeks to determine whether glabridin’s inhibitory effects can be applied before the infection process, potentially providing insights into its mechanism of action against *T. gondii*.

### Inhibitory effect of glabridin on intracellular *T. gondii*

Vero cells were cultured at 37 °C with 5% CO_2_ in six-well plates (5 × 10^5^ cells/well in 2 ml medium). The cells were infected with *T. gondii* RH strain tachyzoites at a MOI of 0.5. After removing free parasites, the cells were treated for 48 h with either 0.1% DMSO (vehicle) or 5 µg/ml glabridin. Genomic DNA was then extracted from the intracellular parasites and host cells. Quantification of intracellular parasites was performed using an absolute qPCR assay described above.

### Assessment of glabridin's impact on *T. gondii* structure

Vero cells were seeded in 12-well culture plates and grown to confluence. Each well was inoculated with 5 × 10^4^ RH tachyzoites and incubated for 8 h. Following infection, the cells were treated with 5 µg/ml glabridin and incubated for 24 or 48 h. After treatment, the medium was discarded, and cells were rinsed twice with phosphate-buffered saline (PBS). The cells and tachyzoites were then fixed with 2.5% glutaraldehyde at 4 °C for 1 h, followed by a PBS rinse and fixation with 1% osmium tetroxide for 1.5 h. The samples were dehydrated through a graded ethanol series, dried at the critical point, coated with gold, and observed under a scanning electron microscope (SEM) to assess the effects of glabridin on the structural morphology of *T. gondii* tachyzoites.

For transmission electron microscopy (TEM), Vero cells were grown in T25 flasks and infected with 2 × 10^6^ *T. gondii* tachyzoites. After 8 h, the medium was replaced with 5 µg/ml glabridin, and cells were incubated for 24 or 48 h. Infected cells were then scraped, centrifuged, rinsed twice with PBS, and fixed with 2.5% glutaraldehyde. Visible clusters of cells and tachyzoites (approximately the size of sesame seeds to mung beans) were collected. These samples were fixed, dehydrated, embedded, and sectioned ultrathin for TEM analysis to examine the effects of glabridin on the internal ultrastructure of *T. gondii*.

### Evaluation of glabridin’s effect on the mitochondria of* T. gondii* tachyzoites

Fresh tachyzoites (2 × 10^6^ per group) were incubated in DMEM containing glabridin (5 µg/ml) for 12 h at 37 °C. Control groups included tachyzoites without drug treatment. Following incubation, JC-1 probe (Beyotime, Shanghai, China) was added and incubated for 20 min. The samples were then rinsed twice with PBS, centrifuged at 1500×*g* for 10 min, and suspended in 200 µl buffer. Fluorescence intensity was observed using fluorescence microscopy, and the JC-1 red-to-green fluorescence intensity ratio was quantified using the multimode reader (SpectraMax® M2e, Molecular Devices, USA).

### Measurement of reactive oxygen species (ROS) production

Tachyzoites (5 × 10^6^ per group) were incubated with 2′,7′-dichlorofluorescein diacetate (DCFH-DA, Solarbio, Beijing, China) for 20 min. Subsequently, 5 µg/ml glabridin or 0.1% DMSO (control) was added for 2 h. The parasites were then washed with PBS, and their fluorescence intensities were measured using a microplate reader (SpectraMax® M2e, Molecular Devices, USA) with excitation and emission wavelengths set at 488 and 525 nm, respectively.

### Sample preparation for metabolomic and transcriptomic analysis

A total of 3 × 10^7^ fresh tachyzoites were added to each of the 6 T75 cell flasks. After 4 h of invasion at 37 °C with 5% CO_2_, the culture medium was divided into two groups. The control group received 2% DMEM without any drug, while the experimental group was treated with 2% DMEM containing 5 µg/ml glabridin. After 24 h of incubation, the cells were scraped and disrupted using a 27G needle. The mixture was then filtered through a 5-µm filter to isolate the parasites, washed twice with PBS, and centrifuged. A total of 1 × 10^6^ tachyzoites were transferred to T25 cell flasks and incubated for 24 h with 2% DMEM, with or without glabridin.

For transcriptomic analysis, three biological replicates per group were used, while six biological replicates per group were used for metabolomic analysis. After rapid freezing in liquid nitrogen, cell lysates were prepared for RNA extraction, RNA sequencing (RNA-seq), and liquid chromatography-tandem mass spectrometry (LC–MS/MS) analysis.

### Transcriptomic sequencing and bioinformatics analysis

Total RNA was extracted using Trizol reagent (Thermo Fisher Scientific, USA, catalog #15596018) following the manufacturer’s instructions. The RNA was fragmented and enriched using oligo (dT) beads for cDNA synthesis, and ribosomal RNA was removed to prepare sequencing libraries. Sequencing was performed on the Illumina NovaSeq™ 6000 platform, generating approximately 1 million 2 × 150 bp paired-end reads. Low-quality reads, adapter sequences, and reads with > 5% unknown bases were removed. Sequence quality was assessed using FastQC (http://www.bioinformatics.babraham.ac.uk/projects/fastqc/, version 0.11.9). High-quality clean reads were aligned to the reference genome using HISAT2 (v2.0.4). Gene expression was analyzed with DESeq2 software, and genes with a false discovery rate (FDR) < 0.05 and an absolute fold change ≥ 2 were considered statistically significant. Gene Ontology (GO) enrichment analysis was conducted using the web-based GO software (http://geneontology.org) [26], and those GO terms showing corrected *P* < 0.05 were considered significantly enriched. Pathway enrichment analysis was conducted using the Kyoto Encyclopedia of Genes and Genomes (KEGG) database (https://www.kegg.jp/kegg/) [27].

### Metabolomic analysis using LC–MS/MS

Metabolomic analysis was conducted by LC-Bio (Hangzhou, China). Metabolites were extracted from samples using an organic reagent precipitation method. Quality control (QC) samples were prepared by combining 10 μl of each extraction mixture for data normalization. The remaining samples were analyzed by LC–MS/MS with electrospray ionization in both positive and negative ion modes. Metabolites were detected using a high-resolution tandem mass spectrometer, the TripleTOF 6600 (SCIEX, Framingham, MA, USA). In positive ion mode, the ion spray voltage was set to 5000 V, and in negative ion mode, it was set to −4500 V. Mass spectral data were acquired in Information-Dependent Acquisition (IDA) mode with a TOF mass range of 60–1200 Da. Raw data were converted to mzXML format using ProteoWizard’s MSConvert software. Peak extraction and quality control were performed using XCMS software. The data matrix, consisting of retention time (RT) and m/z data, was used to identify ions and record peak intensities. Metabolites were annotated by matching exact molecular weight data (m/z) against the KEGG and HMDB databases. Peak intensity data were further processed using MetaX, and relative standard deviations (SDs) of metabolic profiles in QC samples were calculated. Metabolites with relative SDs exceeding 30% were excluded.

### Integrated transcriptomic and metabolomic analysis

To identify key pathways, we integrated transcriptomic and metabolomic data by creating intersecting Venn diagrams. This method enabled us to identify pathways enriched with significantly differentially expressed genes and metabolites. We then focused on analyzing the differential genes and metabolites within these intersected pathways.

### In vivo evaluation of glabridin anti-*T. gondii* effect

Forty female BALB/c mice were divided into four groups (10 mice per group) for treatment. All mice, except those in the mock group, were infected with 100 RH tachyzoites of *T. gondii*. Treatment with glabridin (50 or 100 mg/kg) or vehicle (corn oil) was administered by oral gavage 4 h after tachyzoite inoculation. The mock group received the same volume of corn oil by gavage. Treatment continued for 7 consecutive days, and the mice were monitored daily, being killed once they reached their humane endpoints.

### Statistical analysis

All experiments were performed in triplicate, and data are presented as the mean ± standard deviation (SD). Statistical significance was evaluated using Student's t test or one-way ANOVA followed by Tukey’s post hoc test for multiple comparisons or the log-rank test for survival analysis. A *P*-value < 0.05 was considered statistically significant.

## Results

### Glabridin inhibits *T. gondii* growth in vitro

**Fig. 1:**
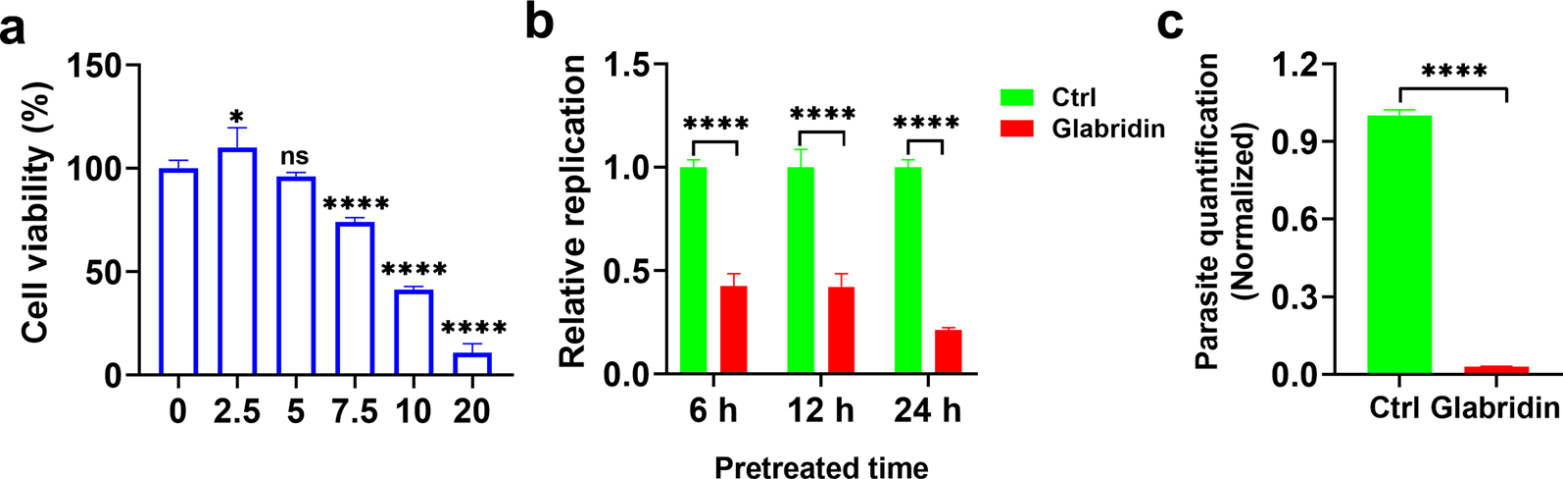
Impact of glabridin treatment on *Toxoplasma gondii* development in vitro and host cell viability. **a** Cytotoxicity of glabridin on Vero cells. Glabridin demonstrated low cytotoxicity in Vero cells. Cells were exposed to increasing concentrations of glabridin (0, 2.5, 5, 7.5, 10, and 20 µg/ml) for 24 h. Cell viability was measured using the Cell Counting Kit-8 (CCK-8) assay, with results expressed as a percentage relative to the untreated control group. Data represent the mean ± SD of three independent experiments. *P < 0.05; ****P < 0.0001. **b** Treatment with glabridin (5 μg/ml) significantly reduced the activity of extracellular *T. gondii* RH tachyzoites. ****P < 0.0001. **c** Inhibitory effect of glabridin (5 μg/ml) on the replication of intracellular *T. gondii* RH tachyzoites. Control samples (Ctrl) were collected 3 h post infection, while treatment groups were collected 48 h post infection, showing a marked decrease in parasite replication. ****P < 0.0001

To assess the toxicity of glabridin on Vero cells in vitro, a CCK-8 assay was performed. The results, shown in Fig. 1a, demonstrated that glabridin exhibited dose-dependent cytotoxicity after 24 h. Specifically, at the concentration of 5 μg/ml, Vero cell growth remained at 99.05% of the control (*P* > 0.05). In contrast, exposure to 7.5 μg/ml of glabridin reduced the growth rate to 74.0% (*P* < 0.0001). Thus, glabridin is considered safe for Vero cells at concentrations ≤ 5 μg/ml.

To evaluate the anti-*T. gondii* activity of glabridin, we examined the proliferation of RH strain tachyzoites treated with 5 μg/ml glabridin or 0.1% DMSO (vehicle). As shown in Fig. 1b, glabridin significantly (*P* < 0.0001) inhibited the extracellular activity of *T. gondii* tachyzoites in a time-dependent manner. Additionally, we assessed its effects on intracellular parasites. Host cells were infected with RH tachyzoites for 3 h, after which extracellular parasites were removed, and fresh media containing either 0.1% DMSO or glabridin was added. After 48 h of culture, glabridin significantly reduced the growth of intracellular *T. gondii* tachyzoites, as shown in Fig. 1c (*P* < 0.0001).

### Effect of glabridin on the ultrastructure of *T. gondii* tachyzoites

**Fig. 2:**
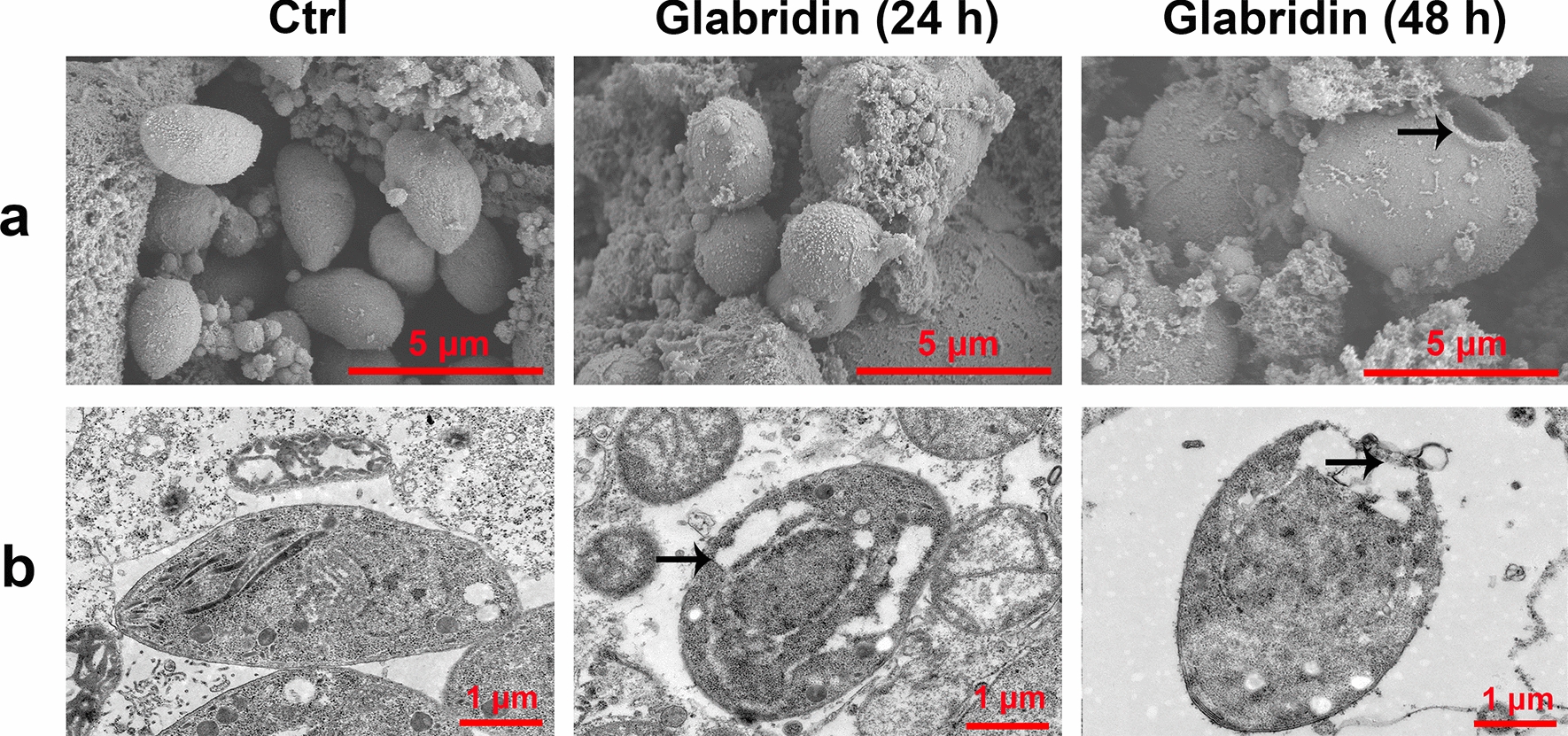
Ultrastructural alterations in *Toxoplasma gondii* tachyzoites induced by glabridin treatment. **a** Scanning electron microscopy and **b** transmission electron microscopy images reveal significant ultrastructural damage in *T. gondii* tachyzoites exposed to 5 μg/ml glabridin. Treated tachyzoites display disrupted plasma membranes, extensive organelle degradation, and large cytoplasmic vacuoles (arrows), indicative of cell death. In contrast, untreated control tachyzoites (Ctrl) show normal morphology with intact organelles

**Fig. 3:**
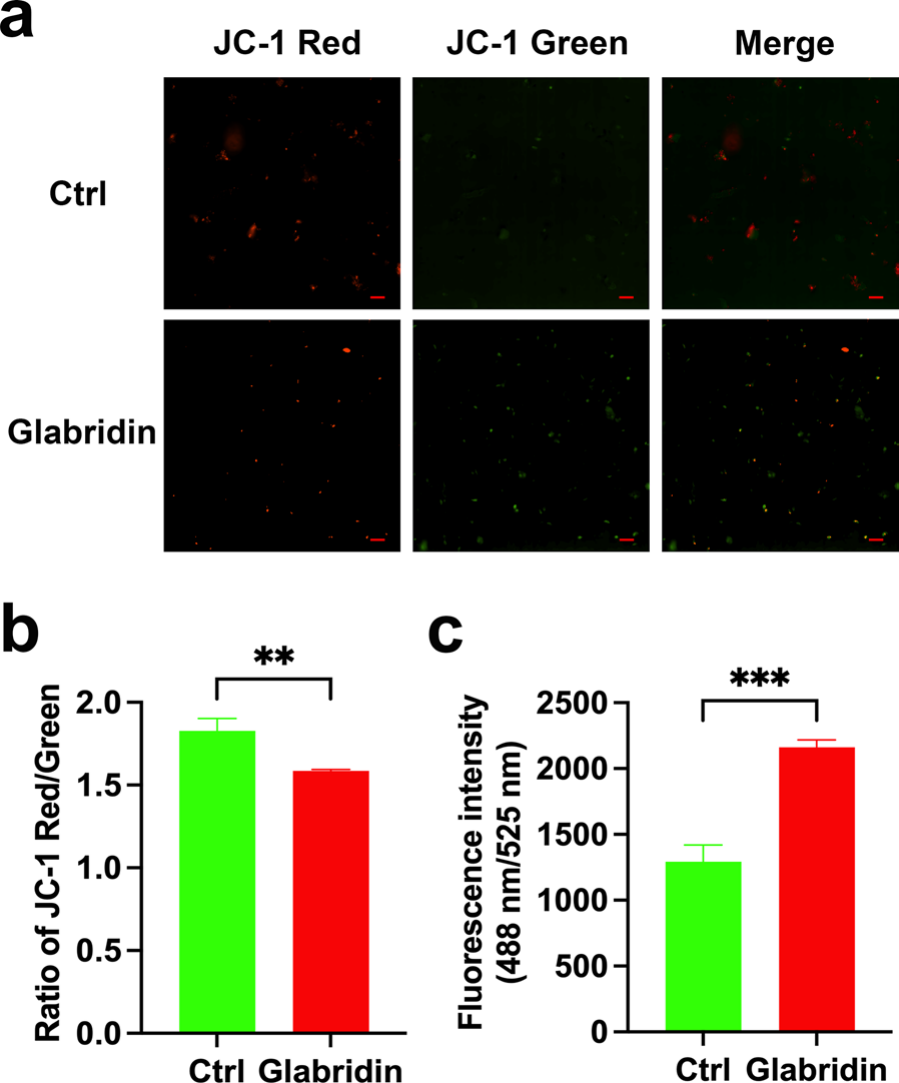
Glabridin treatment decreased mitochondrial membrane potential and increased reactive oxygen species (ROS) production in *Toxoplasma gondii* tachyzoites. **a** Representative fluorescence intensity images for treated and untreated parasites. Scale bars: 20 μm. **b** The JC-1 red-to-green fluorescence intensity ratio of treated and untreated parasites. **P < 0.01. **c** Significant increase in the ROS level observed in the treated parasites. ***P < 0.001

To further investigate the mechanism underlying the anti-*T. gondii* action of glabridin, we examined its effects on the surface and internal ultrastructure of tachyzoites using SEM and TEM. The results are shown in Fig. 2. In the control group, tachyzoites exhibited a smooth surface and normal morphology. After 24 h of incubation with glabridin, the tachyzoite surfaces became concave, twisted, and deformed. After 48 h, the tachyzoites were markedly wrinkled, nearly round, and ruptured. TEM analysis revealed that in the control group, tachyzoites had a characteristic banana-shaped or semilunar longitudinal cross-section with an enlarged center and pointed ends. Key organelles, including mitochondria, nuclei, rhoptries, and dense granules, were clearly visible. After 24 h of glabridin treatment, tachyzoites showed swelling and deformation, with organelles becoming indistinct. After 48 h, the internal organelles were largely absent, the conoid was nearly gone, the membrane system structure was disrupted, and pronounced vacuolation appeared in the cytoplasm. These changes suggest that the tachyzoites were likely dead. The mitochondrial membrane potential (ΔΨm) results showed that glabridin treatment significantly reduced the JC-1 red-to-green fluorescence intensity ratio in the parasites (Fig. 3a, b). The level of ROS in the glabridin-treated parasites was significantly increased compared to untreated parasites (Fig. 3c). These results indicate that glabridin induced membrane damage in *T. gondii*, reduced ΔΨm, and increased ROS production in extracellular tachyzoites.

### Glabridin alters the transcriptome of *T. gondii*

**Fig. 4:**
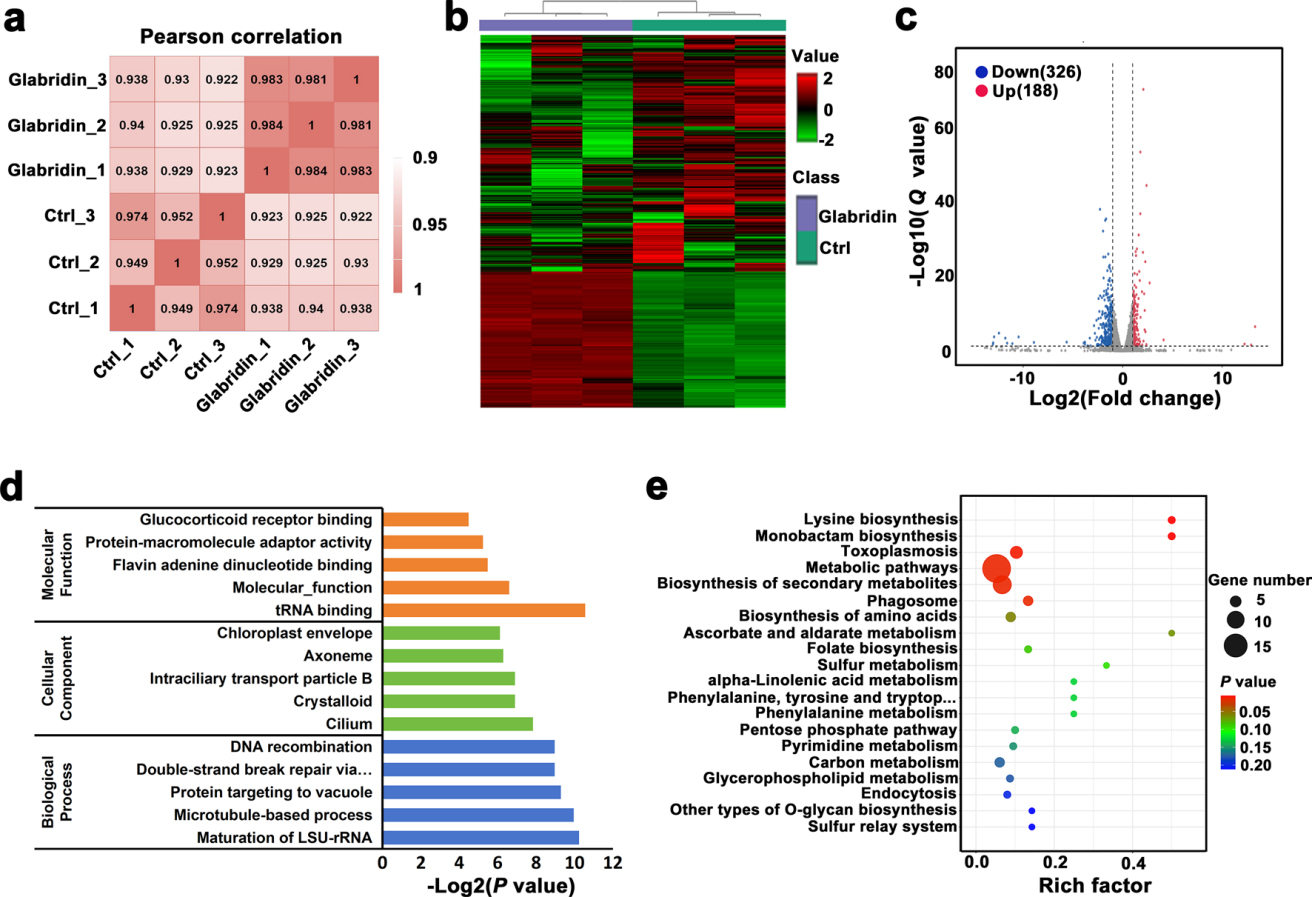
Glabridin-induced transcriptomic changes in *Toxoplasma gondii*. **a** Heatmap of Pearson correlation coefficients between samples. **b** Unsupervised hierarchical clustering of RNA sequencing (RNA-seq) data, with normalized FPKM values (green: low expression, red: high expression). Columns are hierarchically clustered using Pearson correlation as the distance metric. **c** Volcano plot displaying − log10 (*Q* values) versus RNA expression ratio between the glabridin-treated group and control (0.1% DMSO). **d** Gene Ontology (GO) analysis of differentially expressed genes (DEGs), with the X-axis indicating -log10(*P* value) and the Y-axis showing GO terms. **e** Kyoto Encyclopedia of Genes and Genomes (KEGG) pathway analysis of DEGs, where the X-axis represents the enrichment ratio and the Y-axis lists KEGG pathway terms. Dot color indicates *P* value, and dot size reflects the number of DEGs enriched in each KEGG pathway

To assess the impact of glabridin on *T. gondii* gene expression, a transcriptomic analysis was performed. *Toxoplasma gondii* was treated with 5 μg/ml glabridin for 24 h. Both the treated and control groups included three biological replicates each. RNA expression patterns were analyzed using a heatmap of Pearson correlation coefficients. As shown in Fig. 4a, the Pearson correlation matrix revealed clear differences between the glabridin-treated and control groups. Unsupervised hierarchical clustering further distinguishes the treated group from the control, as illustrated in Fig. 4b. Differential expression analysis with DESeq identified 188 upregulated and 326 downregulated genes in the glabridin-treated group compared to controls (Fig. 4c). A detailed list of these differentially expressed genes is provided in Additional File 1: Table S1.

GO enrichment analysis was performed to better understand the functions of the differentially expressed genes. The GO analysis, shown in Fig. 4d, includes biological processes (BP), molecular functions (MF), and cellular components (CC). The most prominent GO terms included tRNA binding, microtubule-based processes, and maturation of LSU-rRNA. Pathway analysis revealed that glabridin significantly affects pathways such as “lysine biosynthesis”, “biosynthesis of secondary metabolites”, and “monobactam biosynthesis” (Fig. 4e).

### Glabridin interferes with *T. gondii* metabolism

**Fig. 5:**
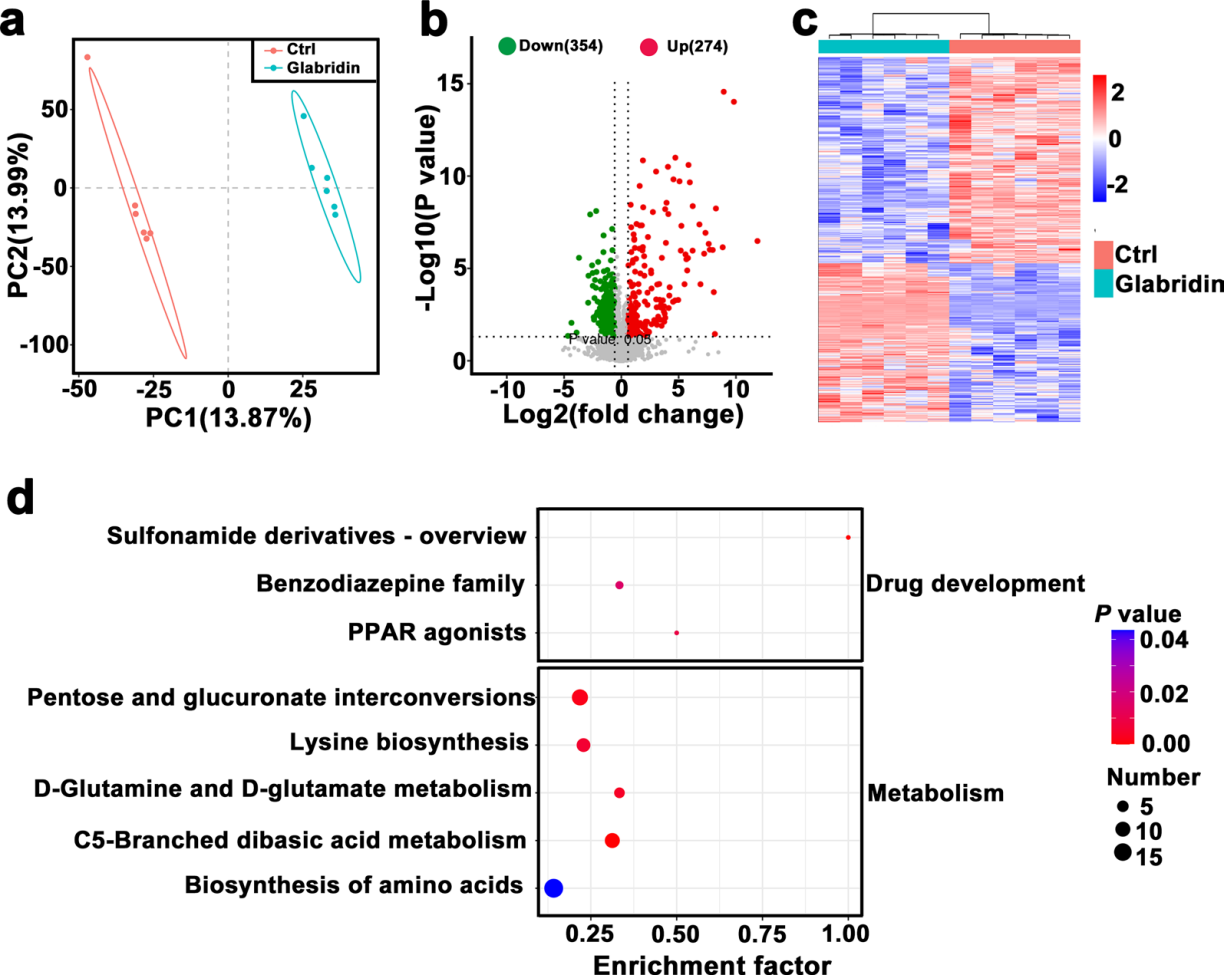
Glabridin’s effects on the metabolomic profile of *Toxoplasma gondii* RH tachyzoites. **a** Partial Least Squares Discriminant Analysis (PLS-DA) illustrating the separation between glabridin-treated and control groups. **b** Volcano plot of differentially accumulated metabolites (DAMs), with log2 fold change on the X-axis and −log10 (*P* value) on the Y-axis. The vertical dotted lines indicate a two-fold change threshold; grey dots represent non-significant metabolites, while red and green dots indicate up- and downregulated DAMs, respectively. **c** Heatmap of DAM clusters, showing metabolic differences between glabridin-treated and untreated *T. gondii*. Red and blue indicate up- and downregulated metabolites, respectively. The impact factor represents the ratio of DAMs to the total metabolites within each pathway. **d** KEGG pathway analysis of differential metabolites, where dot color reflects log10 (*P* value) and dot size corresponds to the number of DAMs enriched in each KEGG pathway

To investigate the effect of glabridin on *T. gondii* metabolism, we employed LC–MS/MS. Differential metabolite accumulation between the treated and control groups was analyzed using Partial Least Squares Discriminant Analysis (PLS-DA) (Fig. 5a). The PLS-DA score plots revealed clear separation between the glabridin-treated and untreated groups in both positive and negative ion modes, indicating significant differences in their metabolite profiles.

In total, 274 metabolites were upregulated, and 354 metabolites were downregulated in the glabridin-treated group compared to controls (Fig. 5b, c). Detailed information on these metabolites is provided in Additional File 2: Table S2. KEGG pathway analysis of differentially accumulated metabolites (DAMs) identified significantly affected pathways, including “biosynthesis of amino acids” and “lysine biosynthesis” (Fig. 5d).

### Effect of glabridin on survival of *T. gondii*-infected mice

**Fig. 6:**
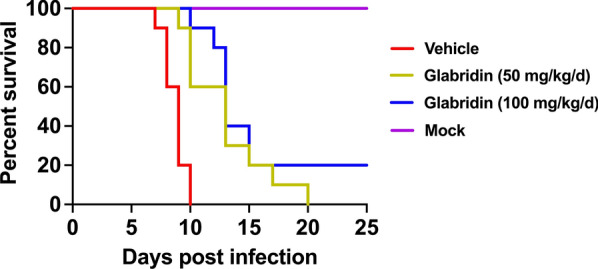
Survival rates of *Toxoplasma gondii*-infected mice treated with glabridin. Mice were intraperitoneally injected with RH tachyzoites and treated with either corn oil (vehicle) or glabridin at doses of 50 or 100 mg/kg. Survival rates were monitored for 25 days. Vehicle mice treated with corn oil reached their humane endpoint on day 7 post-infection. In contrast, mice treated with glabridin at 50 and 100 mg/kg glabridin started to reach their humane endpoints on days 9 and 10, respectively. Treatment with glabridin, particularly at 100 mg/kg dose, significantly delayed the onset of humane endpoints and improved the overall survival rate compared to the vehicle group

To evaluate the in vivo impact of glabridin on *T. gondii*, we assessed the survival rates of infected mice. Mice were intraperitoneally injected with RH tachyzoites and then treated with glabridin. Treatment with glabridin significantly reduced mortality compared to the control group (Fig. 6). Mice infected with *T. gondii* and treated with corn oil reached their humane endpoints starting on day 7 post-infection, with all mice reaching the humane endpoint within 10 days. In contrast, mice receiving 50 or 100 mg/kg glabridin began to reach their humane endpoints on days 9 and 10, respectively. Treatment with 50 or 100 mg/kg glabridin significantly extended the survival time of infected mice, with two mice (20%) treated with 100 mg/kg glabridin surviving until day 25 post-infection.

## Discussion

Natural products have been instrumental in drug discovery, with plant-derived compounds serving as a rich source of therapeutic agents. A significant proportion of commercially available drugs are directly or indirectly derived from plants [28, 29]. Although glabridin has demonstrated various pharmacological activities [30, 31], its potential as an anti-*T. gondii* agent has not been extensively studied. Preliminary work by our group suggested that glabridin exhibits notable in vitro activity against *T. gondii*, justifying further investigation.

In this study, glabridin showed low cytotoxicity in Vero cells and even promoted cell proliferation at specific concentrations. Additionally, glabridin exhibited substantial activity against *T. gondii* RH strain tachyzoites. An in vitro anti-invasion assay demonstrated that glabridin effectively inhibited tachyzoite invasion in a time-dependent manner. To assess glabridin’s impact on *T. gondii* at the cellular level, we conducted SEM and TEM analyses. Results revealed marked alterations in the surface and internal ultrastructure of *T. gondii* following glabridin treatment over various time periods. After 24 h, treated tachyzoites displayed deformation, concavity, and atrophy. By 48 h, severe damage was apparent, including disruption of the double nuclear membrane, dispersed chromatin, cytoplasmic fissures, formation of liposomes, and vacuoles with irregular shapes. The absence of a well-defined nuclear membrane and chromosomal structure suggested cytoplasmic disintegration and initiation of cell death. These findings strongly support glabridin’s significant in vitro anti-*T. gondii* activity.

Prolonged alterations in ΔΨm can inactivate cells and trigger pathological reactions [32]. Mitochondria are also a main source of ROS, which are by-products of aerobic metabolism. ROS include superoxide anion, hydrogen peroxide, and hydroxyl radicals, which play a role in numerous biological processes [33]. Evidence suggests that natural products, such as myrislignan (a main active ingredient of nutmeg [34]) and kijimicin (a natural polyether ionophore originally isolated from the bacterium *Actinomadura* sp. [35]) can reduce *T. gondii* ΔΨm while generating ROS, which collectively inhibit *T. gondii* growth. In this study, we observed that glabridin treatment significantly reduced the ΔΨm of *T. gondii*, disrupted the inner mitochondrial membrane of tachyzoites, and increased ROS production. These findings suggest that glabridin interferes with redox balance and mitochondrial functions of *T. gondii*, ultimately impairing its metabolism and replication.

Integrating transcriptomic and metabolomic analyses allows for the identification of key metabolites and regulatory elements involved in complex biological processes [36]. As an intracellular parasite, *T. gondii* relies heavily on metabolic exchanges with its host [37], with diverse metabolic pathways compartmentalized within various cellular structures, including central carbon metabolism, lipid metabolism, and the synthesis of cofactors and vitamins [38–40]. For example, glycolysis, gluconeogenesis, and fatty acid synthesis occur in the cytoplasm; fatty acid elongation takes place in the endoplasmic reticulum; and the mitochondria facilitate branched-chain amino acid degradation, oxidative phosphorylation, and the TCA cycle [41].

Aspartic acid plays a critical role in biosynthesis, serving as a precursor to lysine and glutamate, which are essential for fat oxidation and protein metabolism. Aspartic proteases are also implicated in *T. gondii*’s development and invasion, aiding in processes such as cell invasion, nutrient uptake, signaling, and immune evasion [42, 43]. Additionally, *T. gondii* synthesizes vital lipids, including fatty acids, phospholipids, and derivatives essential for its survival [44, 45].

In this study, glabridin treatment induced significant changes in *T. gondii*’s transcriptomic and metabolomic profiles. Differential expression analysis identified 514 differentially expressed genes (DEGs) and 628 altered metabolites. KEGG pathway analysis showed significant alterations in pathways related to “lysine biosynthesis”, “biosynthesis of secondary metabolites”, “monobactam biosynthesis”, and “amino acid biosynthesis”. These findings suggest that glabridin may exert its anti-*T. gondii* effects by disrupting key biological processes and interfering with amino acid metabolism.

We further explored glabridin’s potential in a mouse model of acute infection. Mice infected with the virulent *T. gondii* RH strain and treated with glabridin exhibited significantly improved survival rates compared to controls. While the vehicle-treated group began to succumb to infection by day 7, glabridin treatment extended survival, with a 50 mg/kg dose prolonging survival time and a 100 mg/kg dose achieving a 20% survival rate at 25 days post-infection. These findings position glabridin as a promising candidate for further evaluation as a novel anti-*T. gondii* agent.

## Conclusions

This study demonstrates, for the first time to our knowldge, that glabridin exhibits low cytotoxicity to host cells and effectively inhibits *T. gondii* invasion and proliferation in a time-dependent manner. Structural damage observed in treated tachyzoites suggests that glabridin induces parasite death through autophagy and membrane destabilization. Glabridin significantly improved survival rates in *T. gondii*-infected BALB/c mice, underscoring its therapeutic potential. Metabolomic and transcriptomic analyses revealed that glabridin disrupts key metabolic pathways, impairing protein synthesis and energy production, which may reduce the likelihood of resistance—a common challenge in current treatments. While promising, further research is needed to elucidate glabridin’s pharmacokinetics, optimal dosing, and long-term safety, especially in chronic and latent infections. Overall, glabridin’s potent anti-*T. gondii* activity, low toxicity, and unique mechanism of action highlight its potential as a new treatment for toxoplasmosis, contributing to the expanding field of natural product-based drug discovery.

## Supplementary Information

Additional file 1: Table S1. Differentially expressed genes of *Toxoplasma gondii* after glabridin treatment.

Additional file 2: Table S2. Differentially accumulated metabolites of *Toxoplasma gondii* after glabridin treatment.

## Data Availability

The datasets supporting the findings of this article are included within the paper and its supplementary materials. The RNA-seq raw data and metabolomics data have been deposited in the Mendeley data (https://data.mendeley.com/preview/m6df4nzzp4?a=49057024-8a1a-44b6-8d30-b366477d958d, https://data.mendeley.com/preview/sd6zh8jnw7?a=d072bc29-412c-4aba-a4b0-8d8f0a409068 and https://data.mendeley.com/preview/gr4t78hfyd?a=cb68f498-493e-4f70-86bf-48b69620794a).

## Abbreviations

DMEM: Dulbecco’s Modified Eagle Medium
FBS: Fetal bovine serum
CCK-8: Cell Counting Kit-8
qPCR: Quantitative polymerase chain reaction
MOI: Multiplicity of infection
PBS: Phosphate-buffered saline
SEM: Scanning electron microscope
TEM: Transmission electron microscopy
ΔΨm: Mitochondrial membrane potential
ROS: Reactive oxygen species
RNA-seq: RNA sequencing
LC–MS/MS: Liquid chromatography-tandem mass spectrometry
FDR: False discovery rate
GO: Gene Ontology
KEGG: Kyoto Encyclopedia of Genes and Genomes
QC: Quality control
IDA: Information-dependent acquisition
RT: Retention time
SD: Standard deviation
BP: Biological processes
MF: Molecular functions
CC: Cellular components
PLS-DA: Partial least squares discriminant analysis
DEGs: Differentially expressed genes
DAMs: Differentially accumulated metabolites
